# Tetra­kis(diethyl ether)tetra-μ_4_-oxido-octa­kis­(penta­fluoro­phen­yl)octa­zinc

**DOI:** 10.1107/S160053681103697X

**Published:** 2011-09-17

**Authors:** Daniel Franz, Hans-Wolfram Lerner, Michael Bolte

**Affiliations:** a Institut für Anorganische Chemie, J. W. Goethe-Universität Frankfurt, Max-von-Laue-Strasse 7, 60438 Frankfurt/Main, Germany

## Abstract

Mol­ecules of the title compound, [Zn_8_(C_6_F_5_)_8_O_4_(C_4_H_10_O)_4_], are located on a special position of site symmetry 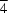. As a result, there is just one quarter-mol­ecule in the asymmetric unit. The title compound features a Zn_4_O_4_ cube. Each Zn atom in the cube carries a pentafluorophenyl substituent. Each O atom is bonded to a further Zn atom, which is connected to a pentafluorophenyl substituent and the O atom of a diethyl ether mol­ecule. All ether C atoms are disordered over two sets of sites with a site occupation factor of 0.51 (2) for the major occupied site.

## Related literature

For background to metal organyls bearing pentafluorophenyl groups, see: Noltes & van den Hurk (1964 ▶); Hayashi *et al.* (2011 ▶); Sun *et al.* (1998 ▶); Weidenbruch *et al.* (1989 ▶). For the chemical shift values of the signals observed in the ^1^H NMR spectrum of free Et_2_O in [D_8_]THF, see: Fulmer *et al.* (2010 ▶).
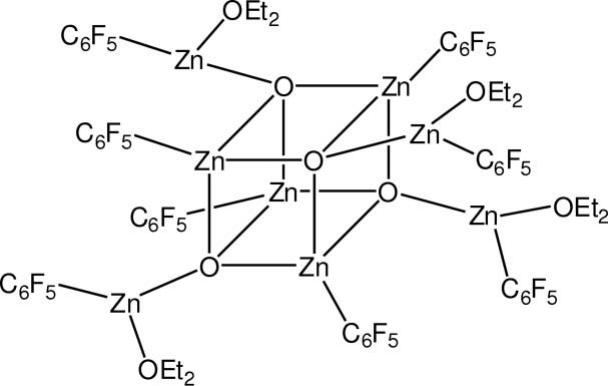


## Experimental

### 

#### Crystal data

[Zn_8_(C_6_F_5_)_8_O_4_(C_4_H_10_O)_4_]
*M* *_r_* = 2219.92
Cubic, 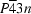

*a* = 23.4948 (6) Å
*V* = 12969.3 (6) Å^3^
*Z* = 6
Mo *K*α radiation
μ = 2.31 mm^−1^
*T* = 173 K
0.35 × 0.33 × 0.32 mm

#### Data collection

Stoe IPDS II two-circle diffractometer
Absorption correction: multi-scan (*MULABS*; Spek, 2009 ▶; Blessing, 1995 ▶) *T*_min_ = 0.498, *T*_max_ = 0.525
75864 measured reflections
4000 independent reflections
3655 reflections with *I* > 2σ(*I*)
*R*_int_ = 0.096

#### Refinement

*R*[*F* ^2^ > 2σ(*F* ^2^)] = 0.038
*wR*(*F* ^2^) = 0.093
*S* = 1.08
4000 reflections
271 parameters
H-atom parameters constrained
Δρ_max_ = 0.57 e Å^−3^
Δρ_min_ = −0.40 e Å^−3^
Absolute structure: Flack (1983 ▶), 1839 Friedel pairs
Flack parameter: −0.002 (19)

### 

Data collection: *X-AREA* (Stoe & Cie, 2001 ▶); cell refinement: *X-AREA*; data reduction: *X-AREA*; program(s) used to solve structure: *SHELXS97* (Sheldrick, 2008 ▶); program(s) used to refine structure: *SHELXL97* (Sheldrick, 2008 ▶); molecular graphics: *XP* in *SHELXTL* (Sheldrick, 2008 ▶); software used to prepare material for publication: *SHELXL97*.

## Supplementary Material

Crystal structure: contains datablock(s) I, global. DOI: 10.1107/S160053681103697X/kj2188sup1.cif

Structure factors: contains datablock(s) I. DOI: 10.1107/S160053681103697X/kj2188Isup2.hkl

Additional supplementary materials:  crystallographic information; 3D view; checkCIF report

## Figures and Tables

**Fig. 1.**
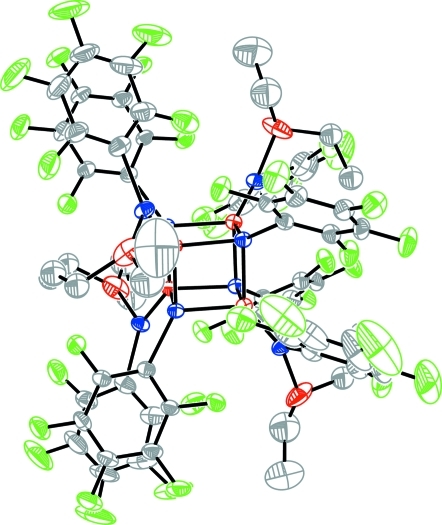
Perspective view of the title compound with displacement ellipsoids at the 30% probability level; H atoms and the minor occupied disordered atoms have been omitted for clarity. Colour codes: C: grey, F: light green, O: red, Zn: blue.

**Fig. 2.**
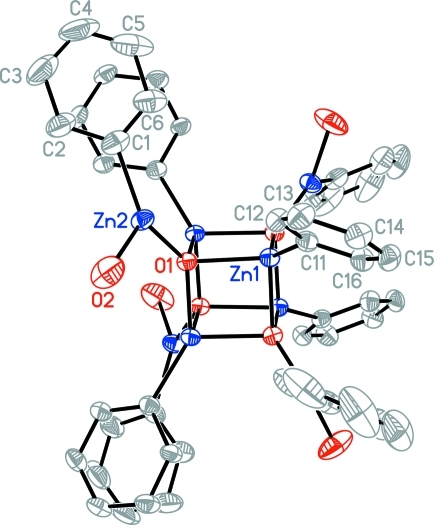
Perspective view of central core of the title compound with displacement ellipsoids at the 30% probability level; Ether C atoms, F and H atoms have been omitted for clarity. Colour codes: C: grey, F: light green, O: red, Zn: blue.

**Fig. 3.**
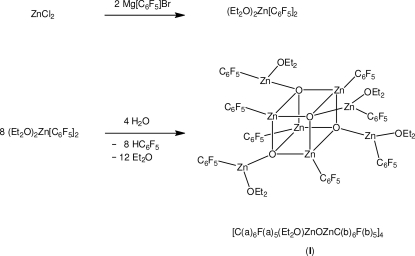
Preparation of the title compound.

## supplementary crystallographic information

### Comment

Since the first synthesis of Zn[C_6_F_5_]_2_ in 1964 many applications of synthesized pentafluorophenyl organyls have been documented (Noltes & van den Hurk, 1964). Despite the long existence and common usage of Zn[C_6_F_5_]_2_, its reactivity towards water has not yet been investigated well. Very recently we have studied the chemical behavior of a series of mesityl derivatives of group 12 elements (Hayashi *et al.*, 2011). It has been shown that the Zn derivative Zn[Mes]_2_ is more reactive towards acids than *M*[Mes]_2_ (*M* = Cd, Hg). In this paper we report the crystal structure of the product which was obtained from the 1 : 1 reaction of (Et_2_O)_2_Zn[C_6_F_5_]_2_ with water. Bis(pentafluorophenyl)zinc was synthesized by slight modification of the method reported in the literature *via* conversion of Mg[C_6_F_5_]Br with ZnCl_2_ in diethyl ether (Noltes & van den Hurk, 1964; Sun *et al.*, 1998). It is interesting to note that the partial hydrolysis of (Et_2_O)_2_Zn[C_6_F_5_]_2_ yields the oxide [C_6_F_5_(Et_2_O)ZnOZnC_6_F_5_]_4_ whereas the 1 : 1 reaction of the corresponding Cd[C_6_F_5_]_2_ with water produces the hydroxy derivative [C_6_F_5_CdOH]_4_ (Weidenbruch *et al.*, 1989). In the solid state the Cd hydroxide [C_6_F_5_CdOH]_4_ also displays a heterocubane structure.

Molecules of the title compound, C_64_H_40_F_40_O_8_Zn_8_, are located on a special position of site symmetry 4. As a result of that, there is just a quarter of a molecule in the asymmetric unit. The title compound features an Zn_4_O_4_ cube. Each Zn atom in the cube carries a pentafluorophenyl substituent. Each O atom is bonded to a further Zn atom which is connected to a pentafluorophenyl substituent and the O atom of a diethyl ether molecule.

### Experimental

All transformations were carried out under an atmosphere of dry nitrogen using Schlenk techniques. Solvents (diethyl ether, toluene) were freshly distilled from sodium/benzophenone  and hexane from sodium prior to use. NMR spectra were recorded on a Bruker Avance 300 (^1^H, ^19^F{^1^H} and a DPX 250 (^13^C{^1^H}). Chemical shift values (^1^H, ^13^C{^1^H}) are reported in p.p.m. relative to SiMe_4_ and were referenced to residual solvent signals. The ^19^F{^1^H} NMR chemical shift values were referenced to external CFCl_3_. The MALDI spectrum was recorded on a FISONS Instruments VG TofSpec using ATT as a matrix. Abbreviations: d = doublet, t = triplet, q = quartet, m = multiplet, br = broad, n.o. = signal not observed.

In a round bottom flask anhydrous ZnCl_2_ (5.40 g, 39.6 mmol) was suspended in diethyl ether (50 ml) and cooled to -30°C. Under stirring, a solution of Mg[C_6_F_5_]Br in diethyl ether (79.4 mmol, 100 ml) was added *via* canula and the mixture was allowed to warm to room temperature over night. Insoluble material was removed by filtration and the solvent was evaporated under reduced pressure. A viscous brown slurry was obtained which was heated to 140°C for a period of approximately 3 h. The residue obtained was dissolved in a 2 : 1 mixture of toluene and hexane and stored at -30°C for 24 h. After the first crystal crop of (Et_2_O)_2_Zn[C_6_F_5_]_2_ had been separated the mother liquor was stored at -30°C for a period of approximately two months yielding crystals of the title compound. This can be attributed to the intrusion of moisture over the period of storage. ^1^H (300.0 MHz, [D_8_]THF, 25°C): δ = 3.39 (q, ^3^*J*_H—H_ = 7.0 Hz, O(*C*H_2_CH_3_)_2_) (the chemical shift values for the signals observed in the ^1^H NMR spectrum equal the values published for free Et_2_O in [D_8_]THF; Fulmer *et al.*, 2010), 1.11 p.p.m. (t, 3*J*_H—H_ = 7.0 Hz, O(CH_2_CH_3_)_2_); ^13^C{^1^H} NMR (62.9 MHz, [D_8_]THF, 25°C): δ = 149.5 (br d, ^1^*J*_C—F_ = 226 Hz, (*o*-C_6_F_5_)-a or b), 149.2 (br d, ^1^*J*_C—F_ = 223 Hz, (*o*-C_6_F_5_)-a or b), 140.5 (br d, ^1^*J*_C—F_ = 258 Hz, (*p*-C_6_F_5_)-a,b), 137.1 p.p.m. (br d, ^1^*J*_C—F_ = 249 Hz, (*m*-C_6_F_5_)-a,b), Et_2_O Signals, n.o. (ZnC); ^19^F{^1^H} NMR (282.3 MHz, [D_8_]THF, 25°C): δ = -111.42 (m, (*o*-C_6_F_5_)-a or b), -114.53 (m, (*o*-C_6_F_5_)-a or b), -156.20 (t, ^3^*J*_F—F_ = 19 Hz, (*p*-C_6_F_5_)-a or b), -156.58 (t, ^3^*J*_F—F_ = 19 Hz, (*p*-C_6_F_5_)-a or b), -161.32 (m, (*m*-C_6_F_5_)-a or b), -161.65 p.p.m. (m, (*m*-C_6_F_5_)-a or b). MALDI+ m/*z* (%): 554.89 (100) [C_6_F_5_(Et_2_O)ZnOZnC_6_F_5_+1]^+^, calcd for [C_6_F_5_(Et_2_O)ZnOZnC_6_F_5_+1]^+^: 554.91 (100).

### Refinement

H atoms were geometrically positioned and refined using a riding model with fixed individual displacement parameters [U(H) = 1.2 *U*_eq_(C) or U(H) = 1.5 *U*_eq_(C_methyl_)] using a riding model with *C*—*H*(methylene) = 0.99Å or *C*—*H*(methyl) = 0.98 Å, respectively.

### Crystal data

**Table:**

[Zn_8_(C_6_F_5_)_8_O_4_(C_4_H_10_O)_4_]	*D*_x_ = 1.705 Mg m^−^^3^
*M**_r_* = 2219.92	Mo *K*α radiation, λ = 0.71073 Å
Cubic, *P*43*n*	Cell parameters from 44493 reflections
Hall symbol: P -4n 2 3	θ = 2.5–25.8°
*a* = 23.4948 (6) Å	µ = 2.31 mm^−^^1^
*V* = 12969.3 (6) Å^3^	*T* = 173 K
*Z* = 6	Block, colourless
*F*(000) = 6528	0.35 × 0.33 × 0.32 mm

### Data collection

**Table:**

Stoe IPDS II two-circle diffractometer	4000 independent reflections
Radiation source: fine-focus sealed tube	3655 reflections with *I* > 2σ(*I*)
graphite	*R*_int_ = 0.096
ω scans	θ_max_ = 25.5°, θ_min_ = 2.5°
Absorption correction: multi-scan (*MULABS*; Spek, 2009; Blessing, 1995)	*h* = −27→28
*T*_min_ = 0.498, *T*_max_ = 0.525	*k* = −18→28
75864 measured reflections	*l* = −28→28

### Refinement

**Table:**

Refinement on *F*^2^	Hydrogen site location: inferred from neighbouring sites
Least-squares matrix: full	H-atom parameters constrained
*R*[*F*^2^ > 2σ(*F*^2^)] = 0.038	*w* = 1/[σ^2^(*F*_o_^2^) + (0.0574*P*)^2^] where *P* = (*F*_o_^2^ + 2*F*_c_^2^)/3
*wR*(*F*^2^) = 0.093	(Δ/σ)_max_ = 0.001
*S* = 1.08	Δρ_max_ = 0.57 e Å^−^^3^
4000 reflections	Δρ_min_ = −0.40 e Å^−^^3^
271 parameters	Extinction correction: *SHELXL97* (Sheldrick, 2008), Fc^*^=kFc[1+0.001xFc^2^λ^3^/sin(2θ)]^-1/4^
0 restraints	Extinction coefficient: 0.00131 (12)
Primary atom site location: structure-invariant direct methods	Absolute structure: Flack (1983), 1839 Friedel pairs
Secondary atom site location: difference Fourier map	Flack parameter: −0.002 (19)

### Special details

**Table:**

Experimental. ;
Geometry. All e.s.d.'s (except the e.s.d. in the dihedral angle between two l.s. planes) are estimated using the full covariance matrix. The cell e.s.d.'s are taken into account individually in the estimation of e.s.d.'s in distances, angles and torsion angles; correlations between e.s.d.'s in cell parameters are only used when they are defined by crystal symmetry. An approximate (isotropic) treatment of cell e.s.d.'s is used for estimating e.s.d.'s involving l.s. planes.
Refinement. Refinement of *F*^2^ against ALL reflections. The weighted *R*-factor *wR* and goodness of fit *S* are based on *F*^2^, conventional *R*-factors *R* are based on *F*, with *F* set to zero for negative *F*^2^. The threshold expression of *F*^2^ > σ(*F*^2^) is used only for calculating *R*-factors(gt) *etc*. and is not relevant to the choice of reflections for refinement. *R*-factors based on *F*^2^ are statistically about twice as large as those based on *F*, and *R*- factors based on ALL data will be even larger.

### Fractional atomic coordinates and isotropic or equivalent isotropic displacement parameters (Å^2^)

**Table:**

	*x*	*y*	*z*	*U*_iso_*/*U*_eq_	Occ. (<1)
Zn1	0.50037 (3)	0.061641 (18)	0.20605 (2)	0.03607 (14)	
Zn2	0.48836 (2)	0.12803 (2)	0.33083 (3)	0.04580 (17)	
O1	0.50255 (15)	0.06020 (12)	0.29312 (12)	0.0371 (6)	
C1	0.4214 (3)	0.1702 (3)	0.3561 (3)	0.0620 (16)	
C2	0.4150 (3)	0.1966 (3)	0.4078 (3)	0.077 (2)	
C3	0.3657 (5)	0.2226 (4)	0.4255 (4)	0.110 (3)	
C4	0.3189 (5)	0.2225 (5)	0.3911 (6)	0.135 (5)	
C5	0.3221 (3)	0.1967 (4)	0.3404 (5)	0.114 (4)	
C6	0.3732 (3)	0.1700 (3)	0.3230 (4)	0.078 (2)	
C11	0.51854 (19)	0.1320 (2)	0.1628 (2)	0.0425 (10)	
C12	0.5218 (2)	0.18472 (19)	0.1880 (2)	0.0441 (11)	
C13	0.5437 (3)	0.2323 (2)	0.1619 (3)	0.0557 (14)	
C14	0.5638 (3)	0.2287 (2)	0.1076 (3)	0.0542 (13)	
C15	0.5611 (3)	0.1768 (3)	0.0799 (2)	0.0552 (13)	
C16	0.5389 (2)	0.1301 (2)	0.1077 (2)	0.0483 (12)	
F2	0.4605 (3)	0.1988 (2)	0.44346 (18)	0.1052 (16)	
F3	0.3626 (4)	0.2482 (3)	0.4764 (3)	0.179 (4)	
F4	0.2699 (4)	0.2452 (4)	0.4065 (4)	0.230 (6)	
F5	0.2763 (2)	0.1940 (3)	0.3044 (4)	0.169 (3)	
F6	0.37461 (18)	0.1443 (2)	0.2707 (2)	0.0906 (13)	
F12	0.5028 (2)	0.19094 (11)	0.24282 (12)	0.0634 (8)	
F13	0.5465 (2)	0.28246 (13)	0.18992 (18)	0.0851 (12)	
F14	0.58640 (19)	0.27394 (15)	0.08172 (19)	0.0782 (11)	
F15	0.5811 (2)	0.17297 (16)	0.02661 (16)	0.0878 (13)	
F16	0.53988 (18)	0.08019 (14)	0.07952 (15)	0.0698 (10)	
O2	0.5635 (2)	0.1608 (3)	0.3571 (3)	0.100 (2)	
C21	0.6146 (17)	0.1819 (17)	0.3404 (17)	0.146 (15)*	0.38 (2)
H21A	0.6265	0.1634	0.3044	0.175*	0.38 (2)
H21B	0.6437	0.1733	0.3697	0.175*	0.38 (2)
C21'	0.5649 (6)	0.2278 (6)	0.3605 (6)	0.082 (4)*	0.62 (2)
H21C	0.5293	0.2433	0.3442	0.099*	0.62 (2)
H21D	0.5671	0.2397	0.4008	0.099*	0.62 (2)
C22	0.6107 (12)	0.2494 (8)	0.3312 (10)	0.265 (12)	
H22A	0.5833	0.2656	0.3583	0.397*	0.38 (2)
H22B	0.5981	0.2575	0.2923	0.397*	0.38 (2)
H22C	0.6482	0.2665	0.3375	0.397*	0.38 (2)
H22D	0.6171	0.2890	0.3428	0.397*	0.62 (2)
H22E	0.6030	0.2480	0.2903	0.397*	0.62 (2)
H22F	0.6447	0.2268	0.3398	0.397*	0.62 (2)
C23	0.5877 (7)	0.1302 (8)	0.4201 (8)	0.079 (5)*	0.49 (2)
H23A	0.6238	0.1099	0.4125	0.095*	0.49 (2)
H23B	0.5959	0.1609	0.4479	0.095*	0.49 (2)
C24	0.5464 (9)	0.0897 (8)	0.4457 (8)	0.089 (6)*	0.49 (2)
H24A	0.5658	0.0663	0.4743	0.133*	0.49 (2)
H24B	0.5307	0.0652	0.4159	0.133*	0.49 (2)
H24C	0.5155	0.1110	0.4639	0.133*	0.49 (2)
C23'	0.6024 (6)	0.1326 (6)	0.3793 (6)	0.069 (5)*	0.51 (2)
H23C	0.6346	0.1576	0.3901	0.083*	0.51 (2)
H23D	0.6165	0.1031	0.3527	0.083*	0.51 (2)
C24'	0.5754 (9)	0.1044 (9)	0.4335 (8)	0.086 (5)*	0.51 (2)
H24D	0.6040	0.0808	0.4526	0.129*	0.51 (2)
H24E	0.5431	0.0806	0.4221	0.129*	0.51 (2)
H24F	0.5622	0.1341	0.4596	0.129*	0.51 (2)

### Atomic displacement parameters (Å^2^)

**Table:**

	*U*^11^	*U*^22^	*U*^33^	*U*^12^	*U*^13^	*U*^23^
Zn1	0.0371 (3)	0.0301 (2)	0.0410 (3)	−0.0003 (2)	0.0001 (3)	0.00158 (19)
Zn2	0.0421 (3)	0.0406 (3)	0.0547 (3)	0.0011 (2)	0.0038 (2)	−0.0109 (2)
O1	0.0337 (14)	0.0367 (14)	0.0410 (14)	0.0009 (14)	0.0006 (15)	−0.0018 (12)
C1	0.066 (4)	0.047 (3)	0.073 (4)	0.006 (3)	0.025 (3)	0.010 (3)
C2	0.085 (5)	0.067 (4)	0.078 (5)	0.028 (4)	0.026 (4)	0.010 (3)
C3	0.137 (9)	0.108 (7)	0.084 (6)	0.046 (6)	0.057 (6)	0.008 (5)
C4	0.109 (8)	0.163 (10)	0.133 (9)	0.086 (8)	0.072 (8)	0.056 (8)
C5	0.059 (5)	0.123 (7)	0.160 (9)	0.030 (5)	0.027 (5)	0.080 (7)
C6	0.062 (4)	0.068 (4)	0.103 (6)	0.012 (3)	0.022 (4)	0.035 (4)
C11	0.042 (2)	0.039 (2)	0.047 (3)	0.0014 (19)	−0.0014 (19)	0.004 (2)
C12	0.052 (3)	0.032 (2)	0.049 (3)	−0.0018 (19)	0.008 (2)	0.0038 (19)
C13	0.066 (3)	0.033 (3)	0.068 (4)	0.000 (2)	0.011 (3)	0.000 (2)
C14	0.059 (3)	0.039 (3)	0.065 (3)	−0.003 (2)	0.013 (3)	0.014 (2)
C15	0.065 (3)	0.055 (3)	0.045 (3)	0.001 (3)	0.014 (2)	0.014 (2)
C16	0.057 (3)	0.037 (3)	0.051 (3)	0.000 (2)	−0.001 (2)	0.005 (2)
F2	0.143 (4)	0.107 (3)	0.066 (3)	0.031 (3)	0.020 (3)	−0.018 (2)
F3	0.243 (9)	0.166 (6)	0.128 (5)	0.095 (6)	0.100 (5)	−0.006 (4)
F4	0.170 (7)	0.269 (10)	0.251 (9)	0.169 (7)	0.136 (7)	0.128 (8)
F5	0.064 (3)	0.209 (7)	0.234 (8)	0.047 (4)	0.016 (4)	0.103 (6)
F6	0.066 (2)	0.094 (3)	0.111 (4)	−0.005 (2)	−0.013 (2)	0.017 (3)
F12	0.093 (2)	0.0402 (14)	0.0566 (16)	−0.0037 (18)	0.024 (2)	0.0002 (12)
F13	0.131 (3)	0.0357 (17)	0.089 (3)	−0.0218 (19)	0.031 (3)	−0.0054 (17)
F14	0.092 (3)	0.0508 (19)	0.092 (3)	−0.0078 (18)	0.029 (2)	0.0174 (18)
F15	0.135 (4)	0.067 (2)	0.061 (2)	0.004 (2)	0.044 (2)	0.0093 (18)
F16	0.110 (3)	0.0468 (17)	0.0532 (18)	−0.0039 (18)	0.0130 (19)	−0.0072 (14)
O2	0.064 (3)	0.113 (4)	0.124 (5)	−0.027 (3)	0.006 (3)	−0.065 (4)
C22	0.39 (3)	0.168 (18)	0.24 (2)	−0.10 (2)	0.00 (2)	−0.021 (16)

### Geometric parameters (Å, °)

**Table:**

Zn1—C11	1.987 (5)	C15—F15	1.339 (6)
Zn1—O1^i^	1.988 (3)	C15—C16	1.379 (7)
Zn1—O1	2.047 (3)	C16—F16	1.347 (6)
Zn1—O1^ii^	2.062 (3)	O2—C23'	1.244 (14)
Zn1—Zn1^iii^	2.8965 (8)	O2—C21	1.36 (4)
Zn1—Zn1^ii^	2.9087 (8)	O2—C21'	1.576 (14)
Zn1—Zn1^i^	2.9087 (8)	O2—C23	1.74 (2)
Zn2—O1	1.854 (3)	C21—C22	1.61 (4)
Zn2—C1	1.951 (6)	C21—H21A	0.9900
Zn2—O2	2.022 (5)	C21—H21B	0.9900
O1—Zn1^ii^	1.988 (3)	C21'—C22	1.37 (2)
O1—Zn1^i^	2.062 (3)	C21'—H21C	0.9900
C1—C2	1.373 (10)	C21'—H21D	0.9900
C1—C6	1.373 (11)	C22—H22A	0.9800
C2—F2	1.359 (9)	C22—H22B	0.9800
C2—C3	1.373 (11)	C22—H22C	0.9800
C3—F3	1.340 (11)	C22—H22D	0.9800
C3—C4	1.365 (17)	C22—H22E	0.9800
C4—F4	1.319 (9)	C22—H22F	0.9800
C4—C5	1.339 (17)	C23—C24	1.49 (3)
C5—F5	1.371 (12)	C23—H23A	0.9900
C5—C6	1.415 (11)	C23—H23B	0.9900
C6—F6	1.370 (10)	C24—H24A	0.9800
C11—C12	1.375 (7)	C24—H24B	0.9800
C11—C16	1.381 (7)	C24—H24C	0.9800
C12—F12	1.373 (6)	C23'—C24'	1.57 (2)
C12—C13	1.374 (7)	C23'—H23C	0.9900
C13—F13	1.352 (7)	C23'—H23D	0.9900
C13—C14	1.363 (8)	C24'—H24D	0.9800
C14—F14	1.335 (6)	C24'—H24E	0.9800
C14—C15	1.385 (8)	C24'—H24F	0.9800
			
C11—Zn1—O1^i^	137.71 (16)	F16—C16—C11	119.6 (4)
C11—Zn1—O1	121.31 (16)	C15—C16—C11	123.3 (5)
O1^i^—Zn1—O1	89.80 (12)	C23'—O2—C21	70.4 (17)
C11—Zn1—O1^ii^	117.83 (16)	C23'—O2—C21'	119.7 (9)
O1^i^—Zn1—O1^ii^	88.69 (11)	C21—O2—C23	96.1 (18)
O1—Zn1—O1^ii^	87.78 (12)	C21'—O2—C23	111.2 (9)
C11—Zn1—Zn1^iii^	146.43 (14)	C23'—O2—Zn2	124.5 (8)
O1^i^—Zn1—Zn1^iii^	45.37 (9)	C21—O2—Zn2	145.4 (18)
O1—Zn1—Zn1^iii^	89.06 (8)	C21'—O2—Zn2	114.4 (6)
O1^ii^—Zn1—Zn1^iii^	43.33 (9)	C23—O2—Zn2	112.8 (7)
C11—Zn1—Zn1^ii^	132.34 (14)	O2—C21—C22	110 (3)
O1^i^—Zn1—Zn1^ii^	89.85 (8)	O2—C21—H21A	109.6
O1—Zn1—Zn1^ii^	43.08 (9)	C22—C21—H21A	109.6
O1^ii^—Zn1—Zn1^ii^	44.72 (8)	O2—C21—H21B	109.6
Zn1^iii^—Zn1—Zn1^ii^	60.137 (9)	C22—C21—H21B	109.6
C11—Zn1—Zn1^i^	151.96 (14)	H21A—C21—H21B	108.1
O1^i^—Zn1—Zn1^i^	44.68 (8)	C22—C21'—O2	111.2 (13)
O1—Zn1—Zn1^i^	45.14 (9)	C22—C21'—H21C	109.4
O1^ii^—Zn1—Zn1^i^	88.44 (8)	O2—C21'—H21C	109.4
Zn1^iii^—Zn1—Zn1^i^	60.137 (9)	C22—C21'—H21D	109.4
Zn1^ii^—Zn1—Zn1^i^	59.724 (18)	O2—C21'—H21D	109.4
O1—Zn2—C1	136.6 (2)	H21C—C21'—H21D	108.0
O1—Zn2—O2	108.4 (2)	C21'—C22—C21	67.1 (16)
C1—Zn2—O2	114.7 (2)	C21'—C22—H22A	46.1
Zn2—O1—Zn1^ii^	137.41 (17)	C21—C22—H22A	109.5
Zn2—O1—Zn1	117.33 (14)	C21'—C22—H22B	107.6
Zn1^ii^—O1—Zn1	92.24 (12)	C21—C22—H22B	109.5
Zn2—O1—Zn1^i^	116.75 (17)	H22A—C22—H22B	109.5
Zn1^ii^—O1—Zn1^i^	91.30 (11)	C21'—C22—H22C	141.3
Zn1—O1—Zn1^i^	90.14 (12)	C21—C22—H22C	109.5
C2—C1—C6	114.3 (6)	H22A—C22—H22C	109.5
C2—C1—Zn2	125.9 (6)	H22B—C22—H22C	109.5
C6—C1—Zn2	119.4 (5)	C21'—C22—H22D	109.5
F2—C2—C3	117.4 (8)	C21—C22—H22D	153.1
F2—C2—C1	118.4 (6)	C21'—C22—H22E	109.5
C3—C2—C1	124.1 (9)	H22C—C22—H22E	109.2
F3—C3—C4	119.0 (9)	H22D—C22—H22E	109.5
F3—C3—C2	121.0 (11)	C21'—C22—H22F	109.5
C4—C3—C2	120.0 (9)	H22D—C22—H22F	109.5
F4—C4—C5	118.4 (14)	H22E—C22—H22F	109.5
F4—C4—C3	122.8 (13)	C24—C23—O2	113.2 (12)
C5—C4—C3	118.8 (8)	C24—C23—H23A	108.9
C4—C5—F5	121.7 (9)	O2—C23—H23A	108.9
C4—C5—C6	120.4 (10)	C24—C23—H23B	108.9
F5—C5—C6	117.9 (11)	O2—C23—H23B	108.9
F6—C6—C1	119.3 (6)	H23A—C23—H23B	107.7
F6—C6—C5	118.4 (8)	C23—C24—H24A	109.5
C1—C6—C5	122.3 (9)	C23—C24—H24B	109.5
C12—C11—C16	114.4 (4)	H24A—C24—H24B	109.5
C12—C11—Zn1	122.8 (4)	C23—C24—H24C	109.5
C16—C11—Zn1	121.8 (4)	H24A—C24—H24C	109.5
C11—C12—F12	118.8 (4)	H24B—C24—H24C	109.5
C11—C12—C13	124.2 (5)	O2—C23'—C24'	105.6 (12)
F12—C12—C13	117.0 (4)	O2—C23'—H23C	110.6
F13—C13—C14	119.5 (5)	C24'—C23'—H23C	110.6
F13—C13—C12	120.7 (5)	O2—C23'—H23D	110.6
C14—C13—C12	119.7 (5)	C24'—C23'—H23D	110.6
F14—C14—C13	121.0 (5)	H23C—C23'—H23D	108.8
F14—C14—C15	120.3 (5)	C23'—C24'—H24D	109.5
C13—C14—C15	118.7 (5)	C23'—C24'—H24E	109.5
F15—C15—C16	121.5 (5)	H24D—C24'—H24E	109.5
F15—C15—C14	118.9 (5)	C23'—C24'—H24F	109.5
C16—C15—C14	119.6 (5)	H24D—C24'—H24F	109.5
F16—C16—C15	117.0 (5)	H24E—C24'—H24F	109.5
			
C1—Zn2—O1—Zn1^ii^	−146.6 (3)	O1—Zn1—C11—C16	154.2 (4)
O2—Zn2—O1—Zn1^ii^	26.8 (4)	O1^ii^—Zn1—C11—C16	48.6 (5)
C1—Zn2—O1—Zn1	84.5 (3)	Zn1^iii^—Zn1—C11—C16	2.3 (6)
O2—Zn2—O1—Zn1	−102.0 (3)	Zn1^ii^—Zn1—C11—C16	101.3 (4)
C1—Zn2—O1—Zn1^i^	−20.8 (4)	Zn1^i^—Zn1—C11—C16	−153.7 (3)
O2—Zn2—O1—Zn1^i^	152.7 (3)	C16—C11—C12—F12	−179.8 (5)
C11—Zn1—O1—Zn2	27.9 (3)	Zn1—C11—C12—F12	−11.1 (7)
O1^i^—Zn1—O1—Zn2	−121.8 (2)	C16—C11—C12—C13	−0.5 (8)
O1^ii^—Zn1—O1—Zn2	149.5 (2)	Zn1—C11—C12—C13	168.2 (4)
Zn1^iii^—Zn1—O1—Zn2	−167.19 (17)	C11—C12—C13—F13	−178.7 (5)
Zn1^ii^—Zn1—O1—Zn2	148.2 (3)	F12—C12—C13—F13	0.6 (8)
Zn1^i^—Zn1—O1—Zn2	−120.5 (2)	C11—C12—C13—C14	0.0 (9)
C11—Zn1—O1—Zn1^ii^	−120.25 (17)	F12—C12—C13—C14	179.4 (5)
O1^ii^—Zn1—O1—Zn1^ii^	1.30 (11)	F13—C13—C14—F14	0.2 (10)
Zn1^iii^—Zn1—O1—Zn1^ii^	44.64 (9)	C12—C13—C14—F14	−178.6 (6)
Zn1^i^—Zn1—O1—Zn1^ii^	91.31 (11)	F13—C13—C14—C15	179.4 (6)
C11—Zn1—O1—Zn1^i^	148.44 (17)	C12—C13—C14—C15	0.6 (9)
O1^i^—Zn1—O1—Zn1^i^	−1.31 (11)	F14—C14—C15—F15	−0.5 (9)
Zn1^iii^—Zn1—O1—Zn1^i^	−46.67 (9)	C13—C14—C15—F15	−179.7 (6)
Zn1^ii^—Zn1—O1—Zn1^i^	−91.31 (11)	F14—C14—C15—C16	178.5 (6)
O1—Zn2—C1—C2	136.8 (5)	C13—C14—C15—C16	−0.7 (9)
O2—Zn2—C1—C2	−36.4 (7)	F15—C15—C16—F16	2.1 (9)
O1—Zn2—C1—C6	−36.3 (6)	C14—C15—C16—F16	−176.9 (5)
O2—Zn2—C1—C6	150.5 (5)	F15—C15—C16—C11	179.3 (6)
C6—C1—C2—F2	179.8 (6)	C14—C15—C16—C11	0.3 (9)
Zn2—C1—C2—F2	6.4 (10)	C12—C11—C16—F16	177.5 (5)
C6—C1—C2—C3	−1.9 (11)	Zn1—C11—C16—F16	8.7 (7)
Zn2—C1—C2—C3	−175.4 (6)	C12—C11—C16—C15	0.3 (8)
F2—C2—C3—F3	−1.0 (13)	Zn1—C11—C16—C15	−168.5 (5)
C1—C2—C3—F3	−179.2 (8)	O1—Zn2—O2—C23'	−43.3 (10)
F2—C2—C3—C4	179.2 (9)	C1—Zn2—O2—C23'	131.7 (10)
C1—C2—C3—C4	0.9 (15)	O1—Zn2—O2—C21	63 (3)
F3—C3—C4—F4	−1.9 (17)	C1—Zn2—O2—C21	−122 (3)
C2—C3—C4—F4	178.0 (9)	O1—Zn2—O2—C21'	150.0 (6)
F3—C3—C4—C5	−179.8 (10)	C1—Zn2—O2—C21'	−35.0 (7)
C2—C3—C4—C5	0.1 (17)	O1—Zn2—O2—C23	−81.5 (7)
F4—C4—C5—F5	0.7 (16)	C1—Zn2—O2—C23	93.6 (7)
C3—C4—C5—F5	178.7 (9)	C23'—O2—C21—C22	−147 (3)
F4—C4—C5—C6	−177.8 (8)	C21'—O2—C21—C22	−12.3 (19)
C3—C4—C5—C6	0.2 (16)	C23—O2—C21—C22	−123 (2)
C2—C1—C6—F6	−179.5 (6)	Zn2—O2—C21—C22	90 (4)
Zn2—C1—C6—F6	−5.7 (8)	C23'—O2—C21'—C22	64.6 (18)
C2—C1—C6—C5	2.2 (10)	C21—O2—C21'—C22	14 (2)
Zn2—C1—C6—C5	176.0 (6)	C23—O2—C21'—C22	102.7 (16)
C4—C5—C6—F6	−179.7 (8)	Zn2—O2—C21'—C22	−128.0 (14)
F5—C5—C6—F6	1.7 (10)	O2—C21'—C22—C21	−12 (2)
C4—C5—C6—C1	−1.4 (13)	O2—C21—C22—C21'	14 (2)
F5—C5—C6—C1	−179.9 (7)	C23'—O2—C23—C24	−123 (2)
O1^i^—Zn1—C11—C12	117.9 (4)	C21—O2—C23—C24	−166 (2)
O1—Zn1—C11—C12	−13.6 (5)	C21'—O2—C23—C24	124.9 (13)
O1^ii^—Zn1—C11—C12	−119.3 (4)	Zn2—O2—C23—C24	−5.3 (15)
Zn1^iii^—Zn1—C11—C12	−165.5 (3)	C21—O2—C23'—C24'	157 (2)
Zn1^ii^—Zn1—C11—C12	−66.6 (5)	C21'—O2—C23'—C24'	107.9 (13)
Zn1^i^—Zn1—C11—C12	38.5 (6)	C23—O2—C23'—C24'	22.7 (14)
O1^i^—Zn1—C11—C16	−74.3 (5)	Zn2—O2—C23'—C24'	−58.2 (15)

Symmetry codes: (i) −*y*+1/2, *x*−1/2, −*z*+1/2; (ii) *y*+1/2, −*x*+1/2, −*z*+1/2; (iii) −*x*+1, −*y*, *z*.
